# Examination of Performance Appraisal Behavior Structure

**DOI:** 10.3389/fpsyg.2016.02075

**Published:** 2017-01-09

**Authors:** Aharon Tziner, Shlomit Levy

**Affiliations:** ^1^Netanya Academic College-Schools of Behavioral Studies and Business AdministrationNeatly, Israel; ^2^Department of Contemporary Jewish Studies, Hebrew University in JerusalemJerusalem, Israel

**Keywords:** attitudes, beliefs, performance appraisal behavior, facet analytic approach, personality, mapping sentence, facet theory

## Abstract

The personality (dispositional) characteristics, attitudes, beliefs, and orientation of 498 managers and military officers toward performance appraisal and organization were collected in order to examine their structural relationships to raters' behavior, in terms of (a) mean appraisal ratings, (b) measures of performance dimensions discrimination, and (c) rate discrimination. A mapping sentence comprising a modality, a reference group, and an aspect (content) facet were used. The empirical results largely confirmed this definitional system. Moreover, a polarizing partition of the space into three regions–Self (rater), Ratee, and Organization/System–was found, possibly implying that these three considerations are equally proximal in determining rater behavior. Future directions for research are advanced.

## Introduction

The rating process has come under much scrutiny in the performance appraisal literature. One of the main conclusions that assists us to understand the nature of that process is that both the appraisal system and its organizational context are critical elements that play a part in the eventual employee evaluation outcomes. These outcomes, in turn, impinge upon the employees' status in the organization and, indeed, on the productivity of the business concern (e.g., Cleveland and Murphy, 1992; Murphy and Cleveland, 1995; Murphy, 2008).

The organizational context covers many aspects of organizational life including (1) raters' personality traits, (2) their attitudes toward the organization, and (3) their beliefs concerning, and attitudes toward, the performance appraisal system. Empirical research has increasingly demonstrated how these dispositions influence raters' performance during the appraisal process (e.g., Tziner et al., 1998; Tziner and Murphy, 1999; Tziner et al., 2002).

### Rater personality

The literature abounds with evidence of the links between broad personality characteristics and behavior in organizations (e.g., Barrick and Mount, 1991; Tett et al., 1991). This body of literature assists us to understand how an employee's attributes and personality traits contribute toward job performance and interaction in groups, among other organizational behaviors. With respect to appraisals, we could expect the traits of conscientiousness and self-monitoring to play a significant role in shaping appraisers' rating behavior, acting both as a direct influence on rating, and as a moderator of the relationships between the rating context and rating behavior.

#### Conscientious raters

Raters who are conscientious are generally dependable, rule-abiding, and diligent. Such conscientious raters are likely to set high standards of performance, duteousness, and motivation to excel on the job (Costa and McCrae, 1992); consequently, they are more likely to conduct their performance rating responsibilities with greater diligence. The result: efficient discrimination among performance appraisal dimensions and among ratees, and less inflation of ratings. Raters who display conscientiousness are less likely to be swayed by the rating context than their less conscientious peers. The possibility thus exists that conscientiousness moderates the relationships between rating context and rating behavior measures (Tziner et al., 2002).

#### Self-monitoring raters

High self-monitoring individuals examine and control their own behavior successfully. They are susceptible to interpersonal and situational cues and typically manifest a strong desire to maximize social approval and to minimize social disapproval (Jawahar and Stone, 1997). Thus, we might also posit that individual differences in self-monitoring are associated with differences in rating behaviors among appraisers. Since social acceptance is a critical factor, in an appraisal context, high self-monitors (in contradistinction to low self-monitors) can be expected to inflate ratings of their subordinates and to discriminate less among ratees and performance dimensions.

### Attitudes toward the organization

Previous studies have confirmed links between organizational climate and rating behavior (e.g., Tziner et al., 2001). In this study we examined, in particular, organizational citizenship, a concept which is defined here as employees' cooperative behavior that is discretionary (rather than compulsory); it is not formally rewarded, and it contributes to the smooth functioning of the organization (Organ, 1988). This “informal” employee behavior has important consequences in the workplace and, indeed, Bolino (1999) has demonstrated how two manifestations of organizational citizenship—employee initiative and proactive cooperation—enhanced organizational functioning. Since high organizational citizenship implies working to promote organizational performance, raters displaying this characteristic can also be expected to pursue their appraisals with greater care. This should manifest itself in less incidents of rating inflation and better discrimination among performance dimensions and ratees.

### Beliefs about the appraisal system

Research conducted by Murphy and Cleveland (1995) and later by Tziner and colleagues (e.g., Tziner et al., 1998; Tziner and Murphy, 1999; Tziner et al., 2002), have borne out the supposition that raters' beliefs about performance appraisal systems are likely to affect their ratings. In particular, raters' beliefs about their ability to carry out the task of performance appraisal (self-efficacy), and the way in which performance appraisals are used in the organization, are important determinants of rating behavior.

#### Self-efficacy

Raters differ in self-efficacy, their concern regarding their competencies with respect to possessing the requisite knowledge, tools, and professional skills with which to appraise their subordinates' performance accurately. In this respect, appraisers are less likely to discriminate among rating dimensions when they feel they lack the information or skills to rate accurately (Tziner et al., 2002).

The way in which appraisers regard themselves in this respect is also likely to play a motivational role that affects both the amount of effort they apply to the task of appraisal and their behavioral choices during that process. Specifically, following Bandura's (1977, 1982) social learning theory, raters' low self-efficacy might induce raters to distort their ratings. Moreover, raters' negative self-perceptions are likely to engender insufficient motivation to come up with appraisals that are solidly grounded, well-documented, reliable, and accurate (Frayne and Latham, 1987). Such adverse consequences have led the researchers to indicate that, under such circumstances, the appraisal process is a futile exercise (Napier and Latham, 1986). In contrast, raters with a high level of self-efficacy might be expected to perform the appraisal task more conscientiously.

#### Ways in which performance appraisal is used

A substantial body of research demonstrates that raters are more likely to be more motivated (Cleveland and Murphy, 1992), lenient (Cleveland et al., 1989; Murphy and Cleveland, 1991; Landy and Farr, 1992) and attentive (Steers and Lee, 1983), when they believe that appraisals are to be used to determine administrative rewards such as promotions or salary raises. In contrast, when ratings are used for feedback purposes, and thus have fewer concrete consequences, supervisors may be more likely to provide biased evaluations. For instance, Fried et al. (1992) demonstrated that when appraisers rated employees who had little experience on the job, or were known to engender low confidence levels in their supervisors and/or the appraisal system, the appraisers were prone to discriminate against these subordinates.

#### Orientation to the appraisal system

Raters' attitudes toward their own work play a crucial part in the way appraisers function during performance evaluations. Some raters are comfortable with the system while others are distrustful and cynical. Raters who have confidence in the results of the appraisal will likely produce more accurate ratings than those who are negative or skeptical. Why, however, would raters adopt a negative attitude to what is, after all, a primary task in their job description?

First, we may cite research conducted by Bernardin and Orban (1985) regarding raters who believed that their colleagues in the organization were biasing their performance appraisals. They perceived the “rogue” appraisers as being too lenient, consequently, inflating their subordinates' ratings to increase the benefits accruing to their workers. As a result of this perception, and especially when the performance appraisals were used for administrative purposes, the initial raters were induced to act likewise and to distort the appraisal results.

Second, raters have been observed as being very uncomfortable when they evaluate subordinates' performance and provide them with feedback (Murphy and Cleveland, 1991, 1995). One consequence of this discomfort is the tendency to inflate the ratings and to avoid making distinctions among subordinates (Villanova et al., 1993). By giving uniformly high appraisals, it appears that the raters avoid the potentially unpleasant consequences of assigning high ratings to some subordinates and low ratings to others.

## Facet analytic approach

In the present study, we capitalized on the facet analytic approach to examine the structure of performance appraisal behavior. After Guttman (1959), this approach posits that the components of a researched issue can be defined formally. Accordingly, the content of a concept is broken down into components or “facets.” We can thus say that a facet is a criterion or a rule to classify items comprising a given concept (Roazzi et al., 2015) and that when we define the structural configuration of a concept, we spell out all its facets, exhausting its content (Elizur, 1984; Tziner, 1987).

We see that this definitional facet approach requires a taxonomy or classification of the content universe under scrutiny. We can thus describe the facets as the most important properties or components of the concept domain (content). The facets, therefore, constitute a classification of the constituents (elements) of a concept's content, according to some rule (i.e., exclusive features).

We are also interested in a taxonomy of the responses of the respondents to the issue under scrutiny. These responses are connected in the form of a statement, called the mapping sentence, which reads like an ordinary sentence (Hackett, 2014). The mapping sentence is then submitted to an empirical investigation, which if substantiated, becomes a valid representation of the content domain. The mapping sentence also serves as the basis of the generation of a theory (Canter, 1985; Shye et al., 1994).

The most compelling evidence of whether the empirical structure of the relationship among the variables conforms to the hypothesized structure appears when the hypothetical topological structure is superimposed onto the SSA depiction. The SSA (Smallest Space Analysis) is the non-metric scaling procedure that portrays geometrically the matrix of intercorrelation among the variables (e.g., questionnaire items and psychological tests). The geometrical display is done so that the intercorrelation among variables, which constitute measures of similarity, are plotted in space by distances between pairs of points: the stronger the intercorrelation the closer will be the points from one another.

The examination of the SSA output begins with an inspection of the inter-correlations matrix. To the extent that the following conditions are fulfilled by all variables, positive or zero inter-correlations are expected to emerge^1^:

1. Variables relate to a common object of exploration (i.e., they concern the same observation or content domain).
2. Variables have the same range of responses (e.g., “5”being very high to “1” being very low) and reflect the same direction (with low figures at one extreme of the range to indicate low preference or disagreement with a statement, and with high figures at the other extreme to indicate high preference or agreement with the statement).
3. The population of the respondents was not selected artificially, specific to the domain of inquiry.

The first principle to be applied is the principle of contiguity which states that the geometric space in the SSA outcome should be partitionable into regions that reflect the facets and their “structs” (i.e., components, elements). According to this principle, variables that share the same facet structs should be more highly correlated and thus closer together in multidimensional space than variables that do not share the same facet structs. For example, in study of the achievement motive, the three variables entitled “preference for tasks involving uncertainty”; “satisfaction with tasks involving uncertainty”; and “undertaking tasks involving uncertainty,” shared the same “structuple” (i.e., a pair of two elements, each comprised in a different facet). Consequently, we would expect them to be closer to each other than to other variables in the space, an expectation that was, in fact, upheld by the empirical data.

Furthermore, the more similar the variables are to each other in terms of their facet structs, the higher their expected inter-correlations. The consequence of this principle is that an inverse relationship is predicted between (a) similarity of variable structuples and (b) their distance within the special representation of their correlations. Indeed, an inspection of the inter-correlation matrix in article reveals that most of the variables that share two structs have a markedly higher inter-correlation than those sharing only one struct.

The division of the structure into regions is accomplished through boundary curves introduced to aggregate the variables according to the structuples of the mapping sentence.

However, variables of a region do not always cluster together. In most studies, the variables employed are only a sample of all conceivable items in the domain of observation. Because they comprise points everywhere in a geometric representation, some variables at the edge of one region may correlate less with other variables of the same region than with certain variables at the edge of neighboring regions.

An important feature of SSA is its relative insensitivity to variations in variable sampling. Thus, two different selections of items from the same observation domain can be expected to result in their small spaces having identical partition patterns. This is true even though the correlation matrices are different. Different correlations lead to considerable variations in variable positioning from one sample to another. Hence, almost identical configurations in the SSA plots can correspond to two considerably different inter-correlation matrices.

### The performance rating context

The rating context factors (e.g., comfort with performance appraisal), rater personality factors (e.g., self-monitoring), and rating behaviors (e.g., the extent of discrimination among ratees) can be classified into three facets: modality of behavior (cognitive, affective, and instrumental); referent group (self, ratee, appraisal system); and aspect (context: personal, interpersonal, organizational). The classification of the present study's variables by the elements comprising these three facets is displayed in Table 1.

**Table 1:** **Classification of the study variables according to elements of the three facets**.

**Variable**	**Modality**	**Reference group**	**Aspect (context)**
Confidence in the appraisal system	Affective	Appraisal system	Organizational
Self-efficacy	Cognitive	Self (rater)	Personal
Use of performance appraisal (between)	Instrumental	Appraisal system	Organizational
Use of performance appraisal (within)	Instrumental	Ratee	Organizational
Comfort with performance appraisal	Affective	Self (rater)	Interpersonal
Organizational citizenship	Instrumental	Self (rater)	Organizational
Conscientiousness	Cognitive	Self (rater)	Personal
Self-monitoring	Cognitive	Self (rater)	Personal
Rating level	Instrumental	Ratee	Interpersonal
Discrimination among ratees	Instrumental	Ratee	Interpersonal
Discrimination among dimensions	Instrumental	Ratee	Organizational

Using these content facets and their elements, we developed the following mapping sentence, which interrelates factors affecting rating behavior:

**Table:** 

Rater (x) in an organization (y) who			
**Facet A: Modality of behavior**			
displays a		$\left\{\begin{array}{l} \begin{array}{l} 1.\text{ Cognitive}\\ 2.\text{ Affective}\\ 3.\text{ Instrumental} \end{array} \end{array}\right\}$	mode of behavior
**Facet B: Reference group**			
in respect to		$\left\{\begin{array}{l} \begin{array}{l} 1.\text{ Self (rater)}\\ 2.\text{ Ratee}\\ 3.\text{ Appraisal system} \end{array} \end{array}\right\}$	in
**Facet C: Aspect (content)**			
a		$\left\{\begin{array}{l} \begin{array}{l} 1.\text{ Personal}\\ 2.\text{ Interpersonal}\\ 3.\text{ Organizational} \end{array} \end{array}\right\}$	context
**Facet D: Range**			
evidences	→	$\left\{\begin{array}{l} \text{High (positive)}\\ \text{Low (negative)} \end{array}\right\}$	rating behavior.

The purpose of the present study was to examine this definitional framework empirically.

## Methods

Rating context factors, raters' conscientiousness, and raters' self-monitoring were measured by means of questionnaires, and were correlated with measures of rating level, discrimination among ratees, and discrimination among rating dimensions.

### Participants and procedure

Questionnaires were distributed to 600 managers from several organizations and to 220 Israeli military officers. All the participants were responsible for appraising the performance of at least five subordinates. Usable data were obtained from 355 managers (59%) and 143 officers (65%). Of the managers, 77.7% were men and 22.3% women, whose average age was 43.54 years (*SD* = 10.01). The average tenure in the current company was 15.03 years (*SD* = 10.46). 11.7% completed high school, 14% had some academic training, while 74.2% held a university degree in fields other than Business Administration. 87.9% officers were male, and 12.1% were female; their average age was 32.31 years (*SD* = 7.02) and tenure in the military, 12.13 years (*SD* = 7.01). 12.6% completed high school, while 5.6% had some academic training, and 81.8% held a university degree.

### Instruments

The instruments were administered in Hebrew and an equivalence of measures was achieved through back translation from English.

#### Rater personality (conscientiousness)

Ten items drawn from the NEO–Five Factors Inventory (Costa and McCrae, 1992) was employed to measure conscientiousness, whereby a high score on the scale reflects a high degree of conscientiousness. The internal consistency was alpha = 0.713 (*M* = 3.66; *SD* = 0.52) for managers, and alpha = 0.83 (*M* = 4.91; *SD* = 0.57) for officers.

#### Self-monitoring

Self-monitoring was gauged by means of five items garnered from an instrument developed by Gangestad and Snyder (1985). A high score on this scale indicates a high level of self-monitoring. The internal consistency of self-monitoring measure was alpha = 0.62 (*M* = 4.42; *SD* = 0.70) for managers and alpha = 0.63 (*M* = 3.83; *SD* = 0.82) for officers.

In regard to all the above measures, it should be noted that they were calculated as average scores from the individual responses to the items comprising each variable.

#### Attitudes toward the organization (organizational citizenship behavior)

Organizational citizenship behavior was assessed using seven items from Podaskoff and MacKenzie's (1989) Organizational Citizenship Behavior Scale. A high score on this measure indicates strong organizational citizenship behavior. The internal consistency of the items used here was alpha = 0.81 (*i* = 5.07; *SD* = 0.74) for managers, and alpha = 0.80 (*M* = 4.99; *SD* = 0.90) for officers.

#### Beliefs about the appraisal system (self-efficacy)

Eight items taken from scales developed by Napier and Latham (1986) were used to measure self-efficacy, specifically to assess the extent to which subjects believed that they possessed the appropriate competencies to appraise their subordinates. A high score indicated a high level of self-efficacy. The internal consistency of this measure was alpha = 0.70 (*M* = 4.67; *SD* = 0.79).

#### Perceptions of uses of performance appraisals

Raters' perceptions concerning the uses of performance appraisal were measured using items drawn from the questionnaire devised by Cleveland et al. (1989), designed to produce two indices, namely: (a) perceptions of the extent to which performance appraisals are used by the organization to distinguish between ratees (between-person discrimination) for administrative purposes, including promotion, remuneration, and the identification of poor performers, and (b) perceptions of the extent to which appraisals are used to identify employees' strengths and weaknesses (within-person discrimination) for such purposes as performance feedback and the identification of individual training needs. Raters who evidenced high scores on these dimensions could be considered as perceiving that their appraisals were extensively used by management to achieve the goals for which the performance appraisals were originally proposed.

The first index consisted of 10 items, and yielded an internal consistency of alpha = 0.863 (*M* = 3.69; *SD* = 0.91) for managers, and alpha = 0.91 (*M* = 3.65; *SD* = 0.99) for officers. The second index consisted of eight items and produced an internal consistency of alpha = 0.69 (*M* = 3.09; *SD* = 0.79) and alpha = 0.75 (*M* = 3.52; *SD* = 0.68) for managers and officers, respectively.

#### Comfort with performance

Eleven items from the Performance Appraisal Discomfort Scale (Villanova et al., 1993) were employed to measure comfort with performance We reversed the responses to the items, in order that a higher score would indicate a stronger degree of comfort with the performance appraisal and feedback. The internal consistency of this measure was alpha = 0.866 (*M* = 5.02; *SD* = 0.73) for managers, and alpha = 0.87 (*M* = 4.93; *SD* = 0.66) for officers.

#### Orientation to appraisal systems

Confidence in the appraisal system was measured using 11 items relating to political considerations taken from Performance Appraisal Questionnaire. The items inquire as to the extent to which political considerations play a role in the process of formulating performance ratings. A high score on this measure indicates that raters perceive the appraisal system to be heavily loaded with political manipulations and distortions and that the appraisers, consequently, harbor low levels of confidence in the appraisal process. The internal consistency of this measure, orientation to appraisal systems, was alpha = 0.82 (*M* = 3.43; *SD* = 0.76) for managers, and alpha = 0.81 (*M* = 3.60; *SD* = 0.79) for officers.

#### Rating behavior measures

Each supervisor rated multiple subordinates (usually three or more) using a 12-item behavioral incident rating scale. The extent to which each behavior was exhibited by the ratee was registered on a 6-point scale ranging from “never”(1) to “always”(6). A high score indicates good performance. The coefficient alpha for these scales was 0.95 for officers and 0.88 for manager.

For each rater, three rating behavior measures were obtained, namely, (1) rating level, (b) discrimination among ratees, and (3) discrimination among dimensions. *Level of rating* was represented by the overall mean of each rater's evaluations. (*M* = 4.57; *SD* = 0.60). Following earlier studies of this nature, the index of ratee discrimination was derived from the standard deviation of the ratee means obtained from each rater (see Tziner et al., 2001; *M* = 0.94; 0.52). Discrimination among dimensions was represented by the variability of the mean score assigned to each performance incident statement by each rater (*M* = 0.76; *SD* = 0.36). It is worth noting that all the scales and measures of this study demonstrated reasonable psychometric qualities in a stream of previous publications (e.g., Tziner et al., 2001).

## Results

The correlation matrix was first computed, Table 2 displays the results.

**Table 2:** **Correlations among study variables (*N* = 498)**.

		**1**	**2**	**3**	**4**	**5**	**6**	**7**	**8**	**9**	**10**	**11**
1	Confidence	100	−07	−11	62	−18	−02	10	−35	−23	05	12
2	Self-efficacy	−07	100	22	13	30	21	41	40	26	08	17
3	Use-between	−11	22	100	09	22	16	24	05	23	00	−31
4	Use-within	62	13	09	100	−28	−18	35	−01	−12	−16	37
5	Comfort	−18	30	22	−28	100	60	16	39	18	21	−29
6	Citizenship	−02	21	16	−18	60	100	10	09	23	13	−28
7	Conscientiousness	10	41	24	35	16	10	100	−12	13	15	45
8	Self-monitoring	−35	40	05	−01	39	09	−12	100	−02	19	39
9	Rating level	−23	26	23	−12	18	23	13	−02	100	−40	−50
10	Discrimination – ratees	05	08	00	−16	21	13	15	19	−40	100	−13
11	Discrimination – dimensions	12	17	−31	37	−29	−28	45	39	−50	−13	100

*Decimals are omitted*.

Inspection of the correlations indicates that the general expectation that the more facet structs the variables share, the higher they will be correlated (according to the contiguity principle) is largely upheld. For example, rating level and discrimination among ratees share the same structuple: Instrumental, Ratee, Interpersonal. Therefore, each of these two variables should inter-correlate higher than either of them with variables with which they have no structs in common, such as self-efficacy, conscientiousness, or self-monitoring (−0.40 vs. 0.26, 0.8, 0.13, 0.15, −0.2, 0.19).

**Figure 1:**
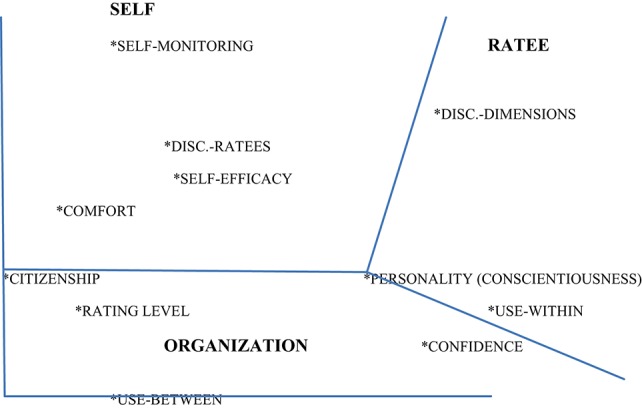
**SSA map of the study variables; coefficient of alienation = 0.104**.

This matrix was then submitted to the SSA software (using a non-metric solution), which maps the variables as points in the Euclidean space of two dimensions. The geometrical configuration is presented in Figure 1.

The coefficient of alienation obtained was 0.104, which can be considered a very good fit of the two-dimensional plot to the original inter-correlation matrix. The figure shows that variables sharing the same facet elements are positioned closer together in the configurative plot than variable that do not.

The division of the space is largely radial (polarizing), with one region consisting primarily of variable related to the self (rater), neighbored by a region of mostly ratee-associated variables, and a third region occupied mainly by the organization/system-related variables.

## Discussion

On the whole, the findings of the present study provide empirical support for the veracity of the mapping sentence, relating rater attitudinal, and dispositional (personality) factors with rating behavior.

Specifically, our results suggest, as in previous investigations and publications (e.g., Tziner et al., 1998, 2001; Tziner and Roch, 2016), that attitudes and beliefs regarding performance appraisal systems and rater personality qualities are relevant factors likely to relate to high (positive) vs. low (negative) rating behaviors. For example, in regard to ratee discrimination, this implies that the rater discriminates between ratees of high vs. low levels of performance by according high ratings to the former and low ratings to the latter. It also emerges from Figure 1 that there is no order between the regions; namely, the rater, the rate, and the organization/system play equal roles in explaining the structure of the considerations and qualities affecting rating behavior. Unlike the study by Tziner et al. (2001), here we cannot conclude that certain factors are more proximal to the task of performance rating than other; all seem to be equally proximal. It is possible that the facet analytic approach helps to discern findings, which remain hidden when linear analytical methods, such as regression analyses, are used.

However, we cannot exclude the possibility that the present results differ from previous findings because of cultural differences. The data in the present investigation were collected from Israeli respondents, whereas previous studies primarily examined North American respondents. Nonetheless, it could well be that the structure generalizes across cultures as it was demonstrated in a study that explored cross-cultural values structure using the facet analytic approach along with the SSA procedure (Gouveia et al., 2015).

We suggest that in order to test the generalizability of the present findings, future studies should be pursued using respondents from various organizations, cultures and organizational strata. Moreover, efforts should directed to exploring whether each facet plays a different role (e.g., modulating, polarizing), and whether the combined interaction results in a defined structure (e.g., radix, conex). The theoretical and practical implications of such structures should be explored.

In summary, the present findings appear encouraging in that they provide clear evidence of the structure of relationships between rater attitudes and beliefs about performance, rater personality qualities, and rating behavior. As such, this study paves the way for further investigations aimed at extending and expanding our understanding of this issue. Likewise, our current study demonstrates as in other OB/HRM investigations, such as the exploration of the coping with stress strategies (Rabenu et al., 2015) and the career span of principal's self-efficacy (Fisher, 2015), that the facet analytic approach along with its statistical tools (e.g., SSA, POSAC) is very instrumental in revealing insights unavailable with other commonly used methodological and statistical procedures.

## Authors' note

The authors wish to thank Gil Sharoni for his help in collecting and analyzing data. and to Professors Kevin R. Murphy and Jeanette N. Cleveland for help with the conception of the theoretical foundation and research instruments.

An earlier version of this paper was presented at the Facet Theory Association Congress.

No ethics review process is needed for survey studies in Israel (both at the national and institutional levels); only in cases where experimental studies with human subjects are pursued such an approval is required.

## Author contributions

All authors listed, have made substantial, direct and intellectual contribution to the work, and approved it for publication.

### Conflict of interest statement

The authors declare that the research was conducted in the absence of any commercial or financial relationships that could be construed as a potential conflict of interest.

## Footnotes

^1^This requirement was termed First Law. First Law is always concerned with the sign of correlation among variables. Second Law calls for an inspection of the relative sizes of correlations, which warrant regional hypothesis derivation.
